# Physiological and transcriptome analyses highlight multiple pathways involved in drought stress in *Medicago falcata*

**DOI:** 10.1371/journal.pone.0266542

**Published:** 2022-04-07

**Authors:** Qian Li, Lili Gu, Jiaxing Song, Chenjian Li, Yanhui Zhang, Yuxiang Wang, Yongzhen Pang, Bo Zhang

**Affiliations:** 1 West Arid Region Grassland Resource and Ecology Key Laboratory, College of Grassland and Environmental Sciences, Xinjiang Agricultural University, Urumqi, China; 2 Institute of Animal Science, Chinese Academy of Agricultural Sciences, Beijing, China; Jawaharlal Nehru University, INDIA

## Abstract

*Medicago falcata* is one of the leguminous forage crops, which grows well in arid and semiarid region. To fully investigate the mechanism of drought resistance response in *M*. *falcata*, we challenged the *M*. *falcata* plants with 30% PEG-6000, and performed physiological and transcriptome analyses. It was found that, the activities of antioxidant enzymes (eg. SOD, POD, and CAT) and soluble sugar content were all increased in the PEG-treated group, as compared to the control group. Transcriptome results showed that a total of 706 genes were differentially expressed in the PEG-treated plants in comparison with the control. Gene enrichment analyses on differentially expressed genes revealed that a number of genes in various pathway were significantly enriched, including the phenylpropanoid biosynthesis (ko00940) and glycolysis/gluconeogenesis (ko00010), indicating the involvement of these key pathways in drought response. Furthermore, the expression levels of seven differentially expressed genes were verified to be involved in drought response in *M*. *falcata* by qPCR. Taken together, these results will provide valuable information related to drought response in *M*. *falcata* and lay a foundation for molecular studies and genetic breeding of legume crops in future research.

## Introduction

Water shortage is one of the most serious abiotic factors affecting plant growth and production, and global climate change threatens sustainable agriculture and may increase the frequency and severity of drought, which in turn prompt to develop drought-tolerant varieties of many crops [1–4]. Plants are very vulnerable to drought stress, and water shortage is fatal to plants and leads to huge economic losses. During the long-term evolution, plants have acquired a series of response protection mechanisms to endure drought stress [5], including morphological, physiological, biochemical and molecular responses. At cellular level, drought signals promote production of stress-protectant metabolites (eg. proline and soluble sugar), trigger the antioxidant system to maintain redox homeostasis, and deploy peroxidase enzymes to prevent acute cellular damage and membrane integrity.

Plants also regulate genes in response to drought stress through the antioxidant system, which could remove the reactive oxygen species (ROS) to relief drought stress [6,7]. In the antioxidant enzyme system, superoxide radicals could be dismutated into H_2_O_2_ and O^2-^ by SOD (Superoxide Dismutase), while H_2_O_2_ was disintegrated by POD (Peroxidase) and CAT (Catalase) [8]. At molecular level, plants under drought stress maintain a certain level of physiological activity through the regulation of thousands of genes and a series of metabolic pathways, in order to reduce or repair damage caused by drought stress [9,10]. Ultimately, physiological and developmental responses to drought are achieved by reprogramming of gene expression and metabolism.

Besides the antioxidant system, many phenylpropanoids and flavonoids were also emphasized to be involved in defense responses to drought stress [8,11]. The phenylpropanoid biosynthetic pathway is activated under drought stress, leading to the accumulation of various phenylpropanoid compounds, which have the potential to remove harmful ROS. Several genes involved in the phenylpropanoid pathway were induced in wheat under drought stress, and the increased tolerance of wheat to drought stress is related to the increase of phenylalanine pathway [8,11,12]. Previous studies on the protemics of the drought stress response in two maize varieties showed that numerous proteins were significantly up-regulated in the glycolysis, which is presumed that expression of these proteins can produce more energy to tolerate drought [13]. However, whether the phenylpropanoid and glycolysis/gluconeogenesis pathway were also involved in drought stress in *Medicago* is still unclear.

*M*. *falcata* is a widely spread legume grass, which is closely related to alfalfa (*Medicago sativa*)—the King of Forage. In comparison, wild *M*. *falcata* exhibits strong tolerance against drought, cold, and soil infertility compared with alfalfa [14,15]. Therefore, *M*. *falcata* was developed as a model legume forage in the studies of abiotic stress responsive mechanisms in legumes [15]. Crossing of *M*. *falcata* with alfalfa generate *Medicago* variety with strong resistance and high utilization value, which makes *M*. *falcata* an important gene bank for alfalfa breeding with resistance traits, and therefore this strategy had been used by alfalfa breeders. In particular, *M*. *falcata* possess high drought tolerance, thus the regulatory mechanisms underlying drought stress in *M*. *falcata* will be useful for the breeding of drought-resistant alfalfa.

In *M*. *falcata*, the variety Wisfal (*Medicago sativa* ssp. *falcata* var. Wisfal) may contribute to the enhanced drought tolerance with higher accumulation of flavonoid antioxidants compared with another variety Chilean (*M*. *sativa* ssp. *sativa* var. Chilean) [14]. Ectopic over-expression of *MfMIPS1* in tobacco increased the activity of MIPS and levels of myo-inositol, galactinol and raffinose, resulting in enhanced resistance to drought [16]. Over-expression of *MfELIP* (Early Light-Induced Protein) gene in tobacco increased tolerance to drought [17]. A lipid-anchored *MfNAC* bound the glyoxalase I (*MtGlyl*) promoter to maintain the glutathione pool in a reduced state under drought stress [18]. *ERN1* and *MfCAS30*/*MtCAS31* were identified to be related to drought response genes in two drought-related subtractive libraries for *M*. *falcata* and *M*. *truncatula* in parallel [19,20]. Furthermore, several transcription factors had been reported to play important roles in response to abiotic stresses including drought [21]. Although these genes from *M*. *falcata* were found to be drought related, the regulation network mechanism on drought-resistance genes are still unclear.

During recent years, RNA-Seq has been explored to dissect the molecular mechanisms of drought tolerance in many plant species [22], including soybean [23], red clover [24], *Agropyron mongolicum* Keng [25] and *M*. *truncatula* [26]. However, studies on drought resistance by using transcriptome technique are rare in *M*. *falcata* [27,28]. *M*. *falcata* lacks genomics resource, partly because it is an autotetraploid, perennial outcrossing species. Thus, study on drought resistance of *M*. *falcata* by RNA-seq is the most critical endeavors to develop necessary tools and integrate molecular breeding approaches in alfalfa. In this study, we explored transcriptome sequencing technique to enrich the transcriptome in *M*. *falcata* and identified genes related to drought resistance. We also screened a number of differentially expressed genes related drought resistance and plant recovery. The physiological responses and transcriptome data of drought resistance can provide information for the breeding of drought resistance in both *M*. *falcata* and alfalfa.

## Materials and methods

### Plant materials and drought treatment

*M*. *falcata* seeds were collected in Xinjiang, and stored at the Grassland Resource and Ecology Key Laboratory of Xinjiang Agricultural University. After physical scarification, seeds were placed on petri dishes and incubated at 4°C for 24h before germinated at 25°C (16h light/8h dark). After germination, seedlings were grown in 3:1:1 topsoil/vermiculite/perlite (v/v/v) mixture at 25°C/18°C (16h/8h day/night). After growth for 1 month, 30% PEG-6000 was added to simulate drought stress, and water was used as control. After one hour PEG treatment, the seedlings (about 60 plants) were harvested as treatment group, and seedlings with water were harvested as control group. The remaining seedlings (about 30) were then subjected to a rehydration treatment for one hour as recovery group. Each group contained a triplicate with more than 3 seedlings for each replicate, 9 samples in total. The whole seedlings (about 2.0g) were immediately rinsed in water, dried with filter paper, and timely placed in liquid nitrogen, and stored at -80°C for RNA extraction and transcriptome analysis (CK1, CK2, CK3 for the control group; T1, T2, T3 for the PEG-treatment group; R1, R2, R3 for the recovery group). Simultaneously, the roots and leaves were further separated, frozen in liquid nitrogen, and stored at -80°C for physiological index measurement, three biological replicates were set for each control group, PEG treatment group and recovery group.

### Measurement of physiological indicators

Half grams of the leaves and root samples were used to quantify relative conductivity as described by Ding and Wang [29], and soluble sugar content by anthrone colorimetry [30]. The activity of antioxidant enzymes including superoxide dismutase (SOD), peroxidase (POD), and catalase (CAT) were assayed using the nitrogen blue tetrazolium (NBT) method [31], the guaiacol method [32], and the UV absorption method [33], respectively. Data were analyzed using one-way ANOVA with software SPSS 20.0.

### RNA extraction, cDNA library construction, and Illumina sequencing

Total RNA was extracted from each sample using RNeasy Plant Mini Kit (Qiagen, Hilden, Germany) following the manufacturer’s protocol. RNA quality was monitored by detection on 1% agarose gels and on an Agilent 2100 Bioanalyzer (Agilent Technologies, CA, USA). mRNA were enriched with the Ribo-Zero^™^ Magnetic Kit (Epicentre, Madison, WI, USA) and the resulting RNA was then used for cDNA library construction with an Ultra RNA Library Prep Kit for Illumina (NEB, Boston, MA, USA) according to the manufacturer’s instructions. cDNA libraries were then sequenced by Denovo Biotechnology Co. (Tianjin, China) using an Illumina HiSeq 2000 sequencer to generate paired-end 150bp (PE150) reads. All sequencing reads were deposited at the Short Read Archive (SRA) of the National Center for Biotechnology Information (NCBI) under the accession number PRJNA625784.

### Sequence analysis, assembly, and annotation

Raw data (reads) in fastq format were firstly processed using an in-house Perl scripts. In this step, clean reads were obtained by removing reads containing adapter sequences, poly-N sequences, or low-quality reads. Then the Q20, Q30, and GC content and sequence duplication were determined, and *de novo* assembly of the transcriptome was carried out using Trinity, with min_kmer_cov value of 2 and all the other parameters were default [34]. Assembly quality was assessed by the length distribution of unigenes. The read counts of unigenes were calculated by the software RSEM v1.2.15 [35], and the gene expression level was calculated using expected number of Fragments Per Kilobase of transcript sequence per Millions base pairs sequenced (FPKM). Plant transcription factor genes were predicted by using iTAK 1.2 software. The basic principle is to use the well-defined transcription factor family genes in the database to identify TF in *M*. *falcata* through hmmscan.

Using RSEM v1.2.15 software, unigenes were mapped with the reference generated by Trinity software, functions of unigenes were annotated by BLAST queries of the NCBI non-redundant protein (Nr) database, the NCBI non-redundant nucleotide sequences (Nt) database, the protein families (Pfam) database, the Swiss-Prot protein database, the Kyoto Encyclopedia of Genes and Genomes (KEGG) Ortholog database, the Gene Ontology (GO) database, and the Clusters of Orthologous Groups of proteins (KOG) database. All queries were performed using a cut-off E-value of 1×10^−5^.

### Gene expression and enrichment analysis

Analysis on differential expression genes of the treatment and control groups was performed using the DEseq R package (1.10.1), and applied with read count values to analyze gene expression levels with a threshold of |log_2_Fold change|≥1 and padj≤0.05. The DEGs were visualized with Venn diagrams and Volcano plots. Clustering was performed using the *pheatmap* R package. GO enrichment analysis of DEGs was carried out using Goseq [36] with corrected *P*<0.05 as threshold. KEGG enrichment analysis of DEGs was performed using KOBAS version 2.0, BH correction for FDR. KEGG pathways with *q* value≤0.05 were considered to be significantly enriched DEGs, and the top 20 pathway entries were presented with enrichment scatter plot.

### qPCR analyses

Quantitative real-time PCR (qPCR) analysis was performed to validate the RNA-Seq results. The primers (S1 Table) were designed for the selected 10 genes using Primer Premier 5.0 Tool, and *actin* gene was used as internal control [37]. Reverse transcription was performed with total RNA using M-MLV Reverse Transcriptase (Promega, Madison, WI, USA). qPCR analysis was conducted using a CFX-96 Real-Time System (Bio-Rad, Hercules, CA, USA), and SYBR Premix Ex Taq (TaKaRa, Kyoto, Japan). qPCRs were performed using the following conditions: denaturation at 95°C for 15 min, followed by 40 cycles of amplification (95°C for 10s, 58°C for 20s, and 72°C for 20 s). Each sample was performed with a technical triplicates, and gene expression was evaluated using the 2−^ΔΔCt^ method.

## Results

### Physiological changes of *M*. *falcata* under drought treatment

**Fig 1:**
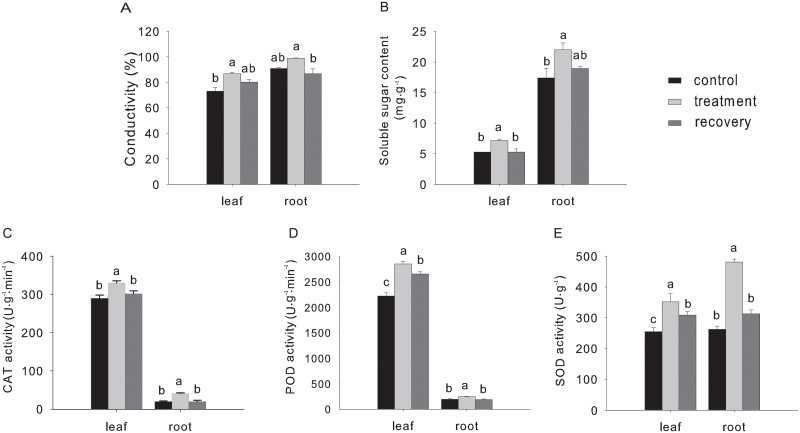
Effect of drought on physiological indexes of *Medicago falcata*. Different lower-case letters represent significant differences between different treatments (*P*<0.05).

We used PEG solution to mimic drought stress, and one-month-old *M*. *falcata* seedlings were treated with PEG solution, with seedlings treated with water as control. Various physiology index from both leaves and roots samples were analyzed from treatment and control seedlings. It was revealed that, under drought stress, conductivity and soluble sugar content were increased significantly (*P*<0.05), whether in leaves or in roots (Fig 1A and 1B). Meanwhile, the enzyme activity of SOD, POD, and CAT were all increased at various level in comparison with the untreated control in both leaves and roots (Fig 1C–1E). After re-watering, seedlings of drought treatment were recovered with comparable level for the majority of physiological indexes (Fig 1), except slight difference for POD and SOD activity in leaves (Fig 1D and 1E). Taken together, these results indicated that drought treatment significantly affected the physiology characteristics of *M*. *falcata*.

### Illumina sequencing and de novo assembly

To further investigate the effect of drought on global transcriptome of *M*. *falcata*, total RNAs were extracted from water control plants, treatment plants and recovery plants (with biological triplicates) and subjected to RNA sequencing. In total, we obtained 559,728,990 raw sequencing reads, and 545,584,982 clean reads were obtained after adaptors and low-quality sequences were removed; the value of Q20 is about 97%, Q30 is about 93%, GC content is more than 40.96% (S2 Table). These high-quality reads were then used to assemble transcriptomic data using Trinity. Assembly of clean reads resulted in 475,378 unigenes (S3 Table), ranging from 201–67,238 bp in size, with an N50 length of 1,026 bp and an N90 length of 263 bp (S4 Table). After comparing different transcripts that represent a single unigene, the longest transcript for each unigene was extracted. After eliminating redundant transcripts, a total of 255,014 unigenes were obtained (S3 Table). Among them, transcripts with length longer than 500 bp accounted for about 37.27%, and unigenes longer than 500 bp accounted for about 33.43% (S3 Table). FPKM is an index that were commonly used to evaluate gene expression levels. We performed statistics on the FPKM values of all samples (S5 Table), and found that FPKM values of less than 3.57 accounted for a large proportion, but the distribution was unstable in each sample. On the contrary, FPKM values of greater than 3.57 showed a stable distribution in all 9 samples. It is possible that the FPKM value was higher, indicating that the gene expression level was relatively higher, and the expression level is relatively stable in the samples (S5 Table).

### Unigenes annotation

**Fig 2:**
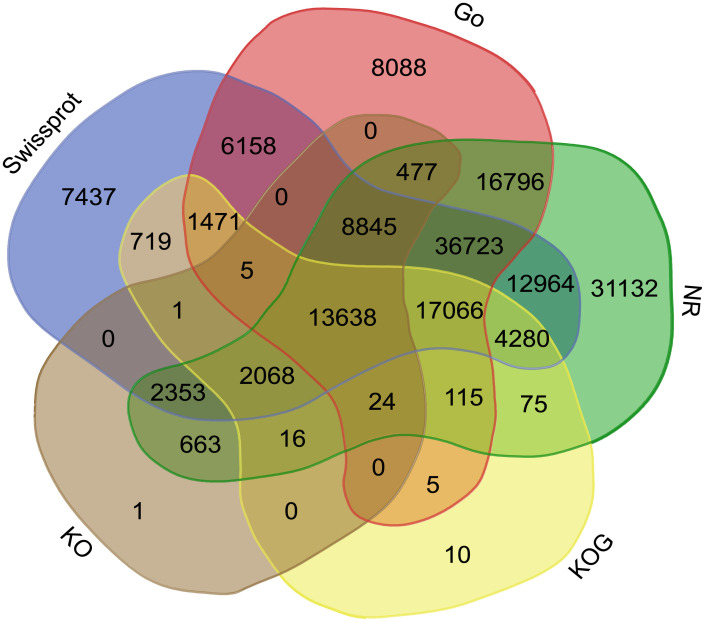
Venn diagram of unigene annotations from the Nr, Swiss-Prot, KOG (EuKaryotic Orthologous Groups), GO (Gene Ontology), and KO (KEGG ortholog) databases.

Venn diagrams of the number of the non-redundant unigenes in GO, NR, KO, KOG and Swiss-Prot databases were constructed to further annotate transcriptome data (Fig 2, S6 Table). It showed that 147,232 (57.73% of the total) and 113,727 (44.59%) unigenes had significant matches in the Nr database and the Swiss-Prot database, and 204,172 (80.06%) had significant matches in the Nt database. We also found that 113,727 (44.59%) non-redundant unigenes showed similarity to known genes in the Swiss-Prot database. In total, 220,137 unigenes (86.32%) were successfully annotated in at least one of the NR, Swiss-Prot, KO, GO, and COG databases (Fig 2 and S6 Table). In order to explore the relationship between *M*. *falcata* and other legumes at transcriptome level, we assembled and compared the *M*. *falcata* transcriptome with *M*. *truncatula*, *Cicer aritinum*, *Glycine max*, *G*. *soja*, *Phaseolus vulgaris* and other legumes in the genome sequence database. A BLASTx comparison of the *M*. *falcata* transcriptome to NCBI nonredundant (nr) peptide database showed that 75.2% gene sequences of *M*. *falcata* (110,621) had significant (e≤1e−5) top hits with those of *M*. *truncatula* (S1 Fig. and S7 Table).

### Functional classification of transcriptome data

**Fig 3:**
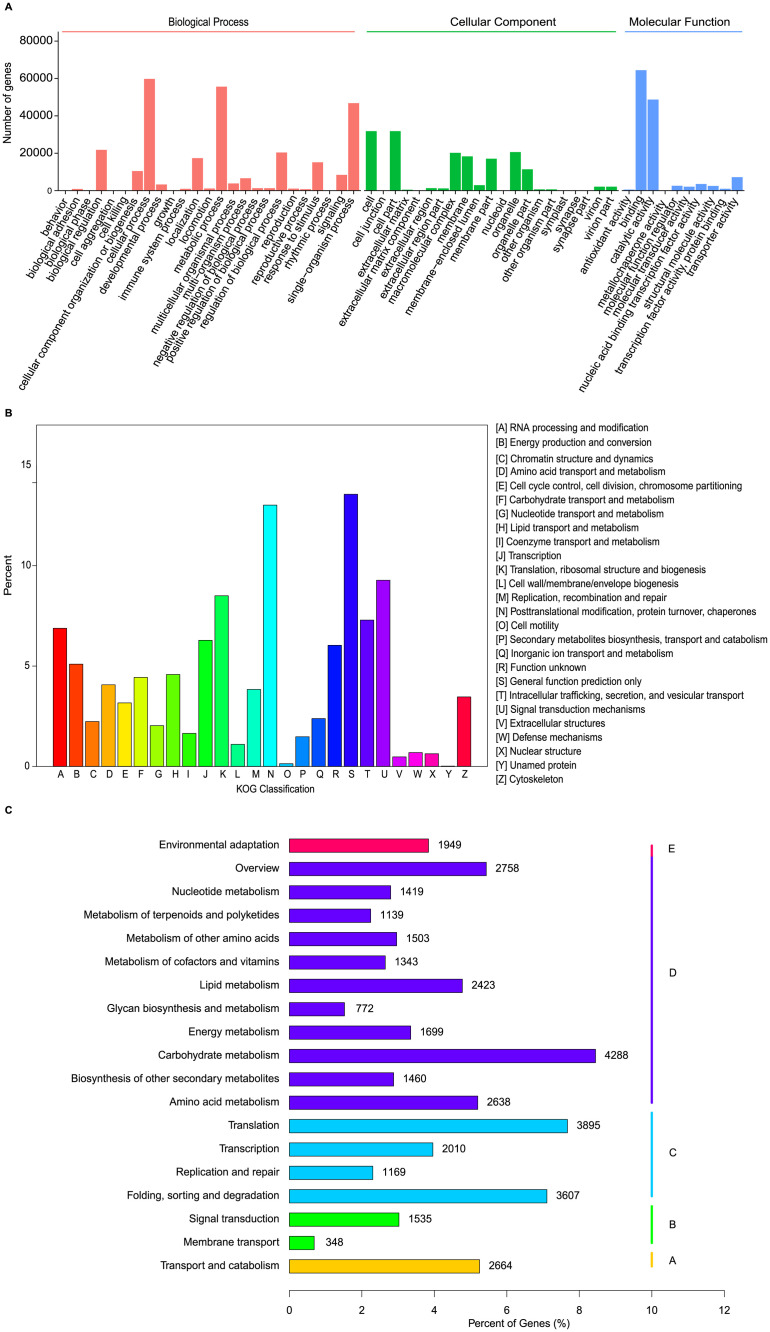
Gene ontology (GO), KOG and KEGG analysis of unigenes. (A) Gene ontology (GO) categorization of unigenes. Different colors represent the three main gene ontology categories; ‘Biological Process’ is indicated in red, ‘Cellular Component’ in green, and ‘Molecular Function’ in blue; (B) KOG function classification; (C) KEGG annotation of unigenes. Different colors represent different KEGG classification, including Cellular Processes (A), Environmental Information Processing (B), Genetic Information Processing (C), Metabolism (D) and Organismal Systems (E).

All transcriptome data were also subjected to three different gene functional classification database, Gene Ontology (GO) database, KOG database and KEGG database. By using Gene Ontology (GO) annotations, total 109,410 non-redundant unigenes were classified into three major functional ontological groups (Fig 3A and S6 Table): biological process, cellular component and molecular function (Fig 3A). Within the category of biological process, the top three subcategories with the most gene numbers were ‘cellular process’ (cellular response to stimulus, cell wall organization or biogenesis), ‘metabolic process’ (hormone, organic substance metabolic process) and ‘single-organism process’ (single-organism metabolic process, developmental process, cellular process) (Fig 3A). Within the cellular component category, genes involved in ‘cell’, ‘cell part’, and ‘organelle’ were highly represented (Fig 3A and S8 Table). Additionally, within the molecular function category, genes related with ‘binding’ and ‘catalytic activity’ were most highly represented (Fig 3A and S8 Table).

Meanwhile, all unigenes were queried against the KOG database for functional prediction and classification. In total 39,492 non-redundant unigenes were subdivided into 26 classifications (Fig 3B and S6 and S9 Tables). Of these, the ‘general function prediction’ cluster (including genetic information processing, metabolism, signaling and cellular processes) was the predominant group, followed by ‘post-translational modification’, ‘protein turnover’, ‘chaperones’, ‘signal transduction mechanisms’, and ‘translation ribosomal structure’ (Fig 3B and S9 Table).

In addition, analysis on metabolic pathway of the unigenes was conducted using the KEGG annotation system. According to the KEGG database, 50,761 unigenes were classed into 5 KEGG first-level classes (Fig 3C and S6 and S10 Tables), including Cellular Processes (A), Environmental Information Processing (B), Genetic Information Processing (C), Metabolism (D) and Organismal Systems (E). Among them, 19 KEGG second-level classes contained 129 distinct pathways, and the pathways with the largest number of unique transcripts were ‘carbohydrate metabolism’ (4,288), followed by ‘translation’ (3,895), and folding, sorting and degradation (3,607) (Fig 3C and S10 Table).

### Analysis on gene expression

**Fig 4:**
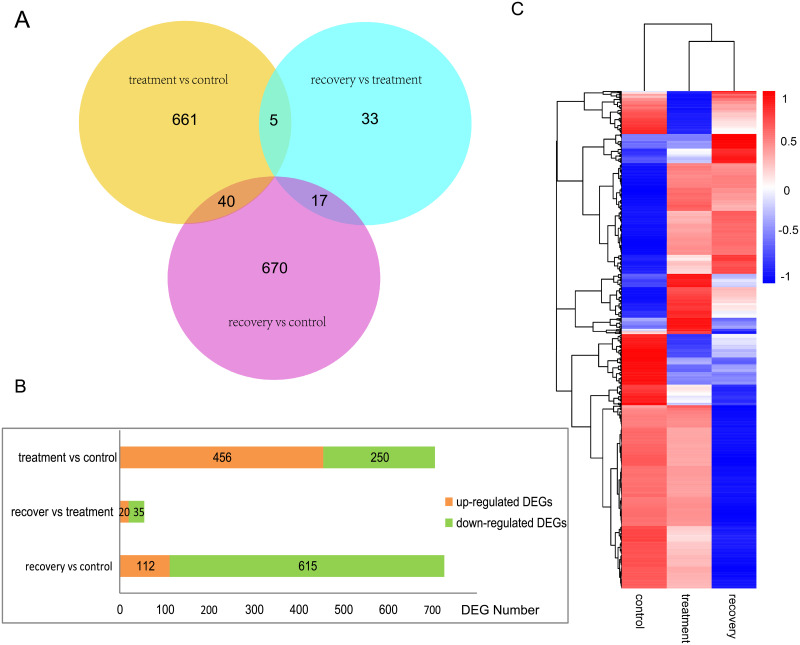
Differential gene expression analysis and clustering. (A)Venn diagram of DEGs. (B) Bar chart showing the number of DEGs detected between treatment combinations. (C) Differential gene clustering; red indicates high levels of gene expression and blue indicates low levels of expression. Colors indicate the range of log_10_ (FPKM+1) values from large (red) to small (blue).

In order to discover new candidate genes that may be involved in drought stress in *M*. *falcata*, we screened genes that were differentially expressed among control, treatment, and recovery plants. Of 255,014 unigenes, 1,426 differentially expressed genes (DEGs) were identified among control, treatment and recovery group (Fig 4A, and S4 Table). Among these DEGs, those with higher expression levels in PEG-treated plants than in control plants were considered to be up-regulated (>2 fold), whereas those with lower expression levels in PEG-treatment were considered to be down-regulated (<2 fold). DEGs with higher expression levels in recovery plants than in PEG-treatment plants were also designated as up-regulated DEGs.

In details, 456 and 250 DEGs were up-regulated and down-regulated respectively in treatment vs control, 20 and 35 genes DEGs were up-regulated and down-regulated respectively in recovery vs treatment, and 112 and 615 genes DEGs were up-regulated and down-regulated respectively in recovery vs control (Fig 4B). By clustering genes with the same or similar expression patterns, the function of the unknown gene or the unknown function of the known gene can be identified, therefore, the expression clustering pattern of all DEGs were determined under different experimental conditions (Fig 4C). Many DEGs showed similar expression pattern in both treatment vs control and recovery vs treatment combinations, including genes encoding Appr1 processing enzyme, 30S ribosomal protein S1, trithorax group protein and ADP-ribosylation factor GTPase-activating protein, indicating that drought stress directly induced the expression of these enzyme genes. Meanwhile, we also found that 10 *POD* genes and one *SOD* gene were differentially expressed among control, PEG-treatment and recovery group (S3 Fig.), in particular, four *POD* genes (Cluster91404, 48742, 17838, and 53806) were up-regulated by PEG-treatment, and they might be the key genes involved in drought resistance in *M*. *falcata*.

### Gene enrichment analysis of DEGs

Gene ontology classification and functional enrichment were performed for the DEGs of three treatment combinations. GO classification and functional enrichment analysis was performed for DEGs from all three treatment combinations. A total of 29 (in 2,090 DEGs), 0 (in 0 DEGs), and 118 (in 6,219 DEGs) GO terms were enriched in DEGs from the treatment vs control, recovery vs treatment, and recovery vs control comparisons, respectively (S8 Table). We identified eight cell-related terms in the cellular component category (GO:0044421, GO:0005622, GO:0044464, GO:0005623, GO:0005576, GO:0005615, GO:0044424, and GO:0043229), as well as two ribosome-related terms (GO:0005840 and GO:0030529), three respiratory chain-related terms (GO:0070469, GO:0045277, and GO:0098803), two cytoplasm-related terms (GO:0044444 and GO:0005737), two organelle-related terms (GO:0043226 and GO:0043228), two growth-related terms (GO:0036454 and GO:0016942) and one macro-molecular complex term (GO:0032991) in the recovery vs control comparison (S11 Table). There was no significant GO functional enrichment terms in the treatment vs control and recovery vs treatment comparisons. However, in the recovery vs control comparison, we identified enriched DEGs in the cell membrane, suggesting that membrane-related genes preferentially responded to drought tolerance.

Several enriched GO terms related to molecular function were identified, including six binding-related terms (GO:0043169, GO:0046872, GO:0043167, GO:0020037, GO:0046906, and GO:0019825), three enzyme activity-related terms (GO:0016702, GO:0016165, and GO:0003953) in the treatment vs control comparison (S11 Table). We also identified fifteen enzyme activity-related terms, five binding-related terms, and ribosomal structural constituent terms in DEGs from the recovery vs control comparison. With respect to biological processes, enriched GO terms related to development, nodulation, and metabolism were identified in the treatment vs control comparison. Enriched GO terms related to biogenesis, metabolic processes, translation (GO:0006412), cell growth (GO:0016049), carbon utilization (GO:0015976), and gene expression (GO:0010467) were also identified in the recovery vs control comparison (S11 Table). No enriched GO terms were identified in the recovery vs treatment comparison. Taken together, these results suggested that the DEGs in response to drought treatment were more complex during the treatment stage, which included oxidoreductase activity, oxygen-, ion-, and cation binding functions, and oxylipin, alanine, and pyruvate family amino acid metabolism-related genes. Finally, at the recovery stage, DEGs were found to be enriched in oxidase and enzyme inhibitor activity-related GO terms, suggesting that plants showed physiological adaptations to drought.

**Fig 5:**
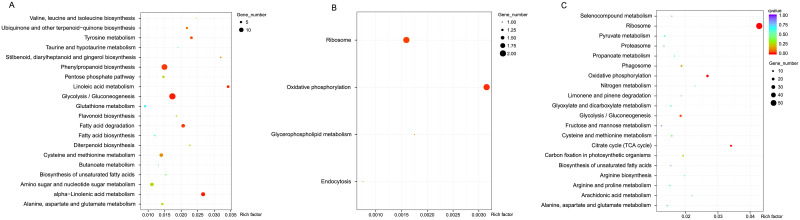
KEGG enrichment analysis of DEGs from the three treatments. (A)Treatment vs control; (B) Treatment vs recovery; (C) Recovery vs control. The vertical axis contains multiple pathways, and the horizontal axis represents the enrichment factor corresponding to individual pathways. The size of the *q-value* is represented by the color of the dot, with smaller *q-*values depicted in red and larger *q-*values in blue. The number of differential genes contained in each pathway is represented by the size of the dot.

To further understand the pathways that the DEGs were involved in, we performed KEGG enrichment analysis with the three different treatments. In total, 20, 4, and 20 enriched pathways were found in the comparisons for treatment vs control (113 DEGs), recovery vs treatment (6 DEGs), and recovery vs control (182 DEGs), respectively (Fig 5, S12 Table). Among them, the glycolysis/gluconeogenesis (ko00010), phenylpropanoid biosynthesis (ko00940), alpha-linolenic acid metabolism (ko00592), fatty acid degradation (ko00071), linoleic acid metabolism (ko00591), tyrosine metabolism (ko00350), ubiquinone and other terpenoid-quinone biosynthesis (ko00130), and flavonoid biosynthesis (ko00941) pathways were found to be significantly enriched in the treatment vs control comparison (Fig 5A and S12 Table).

In the comparison between recovery and treatment, only 3 significantly enriched pathways were identified, including the oxidative phosphorylation (ko00190), ribosome (ko03010) and glycerophospholipid metabolism (ko00564) (Fig 5B and S12 Table). In comparison with the recovery and control, we identified eight significantly enriched pathways, including ribosome (ko03010), oxidative phosphorylation (ko00190), citrate/TCA cycle (ko00020), glycolysis/gluconeogenesis (ko00010), phagosome (ko04145), carbon fixation in photosynthetic organisms (ko00710), cysteine and methionine metabolism (ko00270) and nitrogen metabolism (ko00910) (Fig 5C and S12 Table). In summary, these results indicated that glycolysis, ribosome, sugar, phenylpropanoid biosynthesis, and linoleic acid metabolic functions are involved in drought induced pathways in *M*. *falcata*.

### Transcript factor genes that were induced under PEG treatment

**Fig 6:**
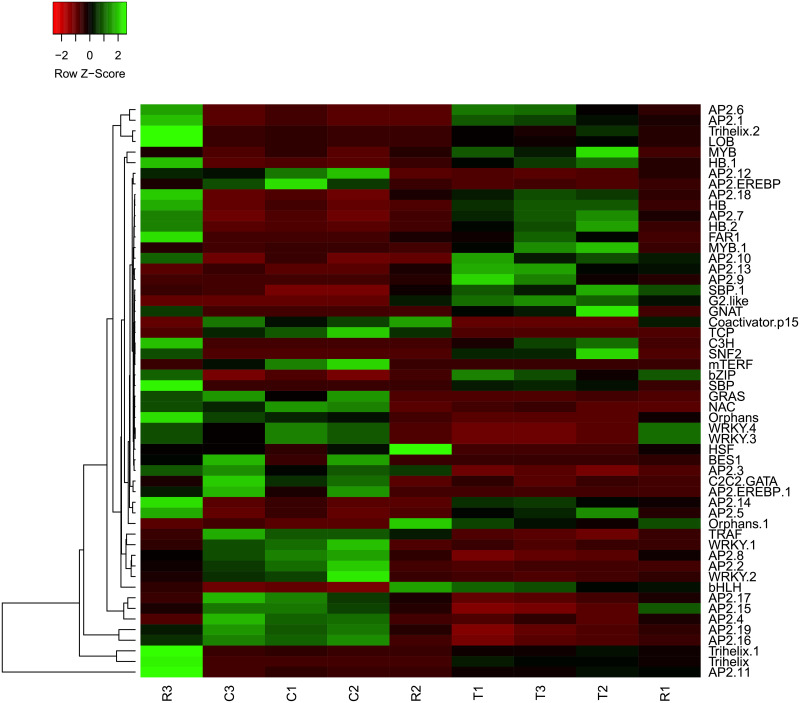
Transcription factor clustering heat map (*P*<0.05). Colors indicate the range of log_10_ (FPKM+1) values from small (red) to large (blue).

Drought stress also induced the expression of many transcription factor genes in *M*. *falcata* (S13 Table). Our results showed that the expression levels of many transcription factor genes, including *C2H2* (594), *MYB* (489), *Orphans* (423), *bHLH* (363), *AP2-EREBP* (358), *bZIP* (278), *C3H* (268), *WRKY* (258), *HB* (253), *PHD* (218), *FAR1* (217), *NAC* (205) and *ABI3VP1TF* (202) families genes were induced at various level. Among them, fifty-four of them were significant expressed in the treatment vs control, three in the recovery vs treatment, and thirty in the recovery vs control (S14 Table). In addition, expression levels of 54 TFs in treatment vs control were represented by FPKM value (Fig 6 and S14 Table), including *AP2*, *MYB*, *WRKY*, *NAC*. The AP2-related TF families was the largest families responded to drought stresses identified in *M*. *falcata*, which accounted for about one-third of the total number, indicating that AP2 family is directly related to drought stress response.

### Drought affects phenylpropanoid and glycolysis/gluconeogenesis pathway

**Fig 7:**
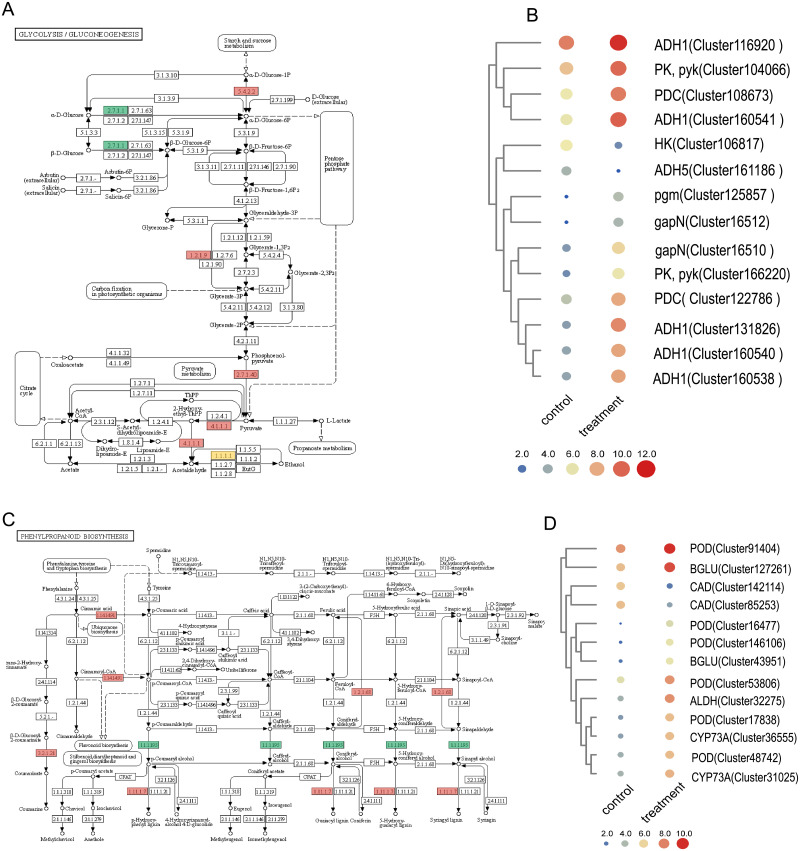
Phenylpropanoid and glycolysis/gluconeogenesis pathway related DEGs. (A) Map of glycolysis/gluconeogenesis pathway; (B) Gene clustering heat map participating in the glycolysis/gluconeogenesis pathway; (C) Map of phenylpropanoid pathway; (D): Gene clustering heat map involving in the phenylpropanoid pathway.

When comparing the gene expression level between the treatment and control groups, we found many genes in the glycolysis/gluconeogenesis pathway were affected. Among them, genes encoding alcohol dehydrogenase class-P (ADH1), pyruvate kinase (PK, phosphoglucomutase), glyceraldehyde-3-phosphate dehydrogenase (gapN), pyruvate decarboxylase (PDC) were up-regulated, and hexokinase (HK) and S-(hydroxymethyl) glutathione dehydrogenase/alcohol dehydrogenase (frmA, ADH5, adhC) were down-regulated (Fig 7A and S15 Table), which may result in abundant production of ATP and NADPH.

It was also found that several genes involved in phenylpropanoid pathway were distinctly regulated when comparing treatment group with control group. The genes encoding peroxidase (POD), coniferyl-aldehyde dehydrogenase (ALDH), trans-cinnamate 4-monooxygenase (CYP73A), and beta-glucosidase (BGLU) were up-regulated, but cinnamyl-alcohol dehydrogenase (CAD) was down-regulated (Fig 7B and S15 Table).

### Validation of differentially expressed genes by qPCR

To further validate the transcriptome data, we selected the top 20 genes (up-regulated and down-regulated) with significant differences in each treatment for cluster analysis (S2 Fig). In the treatment vs control comparison, genes encoding NCR peptides, EHBP1 proteins, E3 ubiquitin-proteins, and early nodulin proteins were significantly up-regulated, while genes encoding Na^+^/H^+^ antiporters, auxin-induced proline rich proteins, ferredoxin-NADP reductase, calmodulin binding protein and ethylene-responsive transcription factors were significantly down-regulated (Fig 5A). In the recovery vs treatment comparison, genes encoding cell wall proteins and NADPH oxidases were significantly up-regulated, while genes encoding ethylene-responsive transcription factors and resistance-like proteins were down-regulated. These results indicated that many genes encoding proteins and transcription factors may function together to coordinate drought stress response.

**Fig 8:**
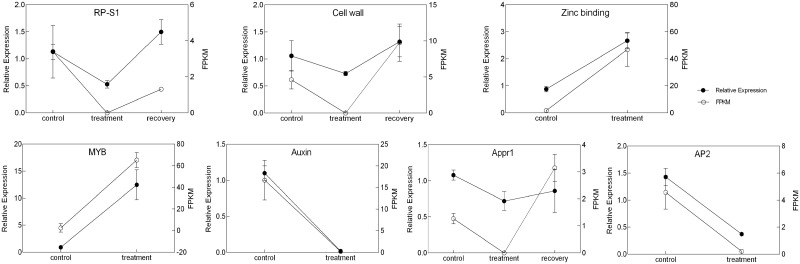
Relative patterns of gene expression by RNA-seq (shown in dark line) and qPCR (shown in gray line). The left coordinate axis shows the FPKM value of transcriptome data, and the right coordinate axis is the relative expression value as per qPCR.

Furthermore, another 10 genes related to drought stress were also selected for verification by qPCR (Fig 8 and S16 Table). Among them, 5 genes were co-expressed as shown in the venn diagram (Fig 4A). The other 5 genes were randomly selected from those that were significantly regulated in the treatment and control combinations, which included genes encoding Myb/SANT-like DNA-binding domain protein, tocopherol cyclase, zinc-binding alcohol dehydrogenase family protein, auxin induced proline rich protein and ethylene-responsive transcription factor ERF110. Among these ten genes that were analyzed by qPCR, seven of them were verified to have the same expression pattern as from the transcriptome data. In particular, the expression levels of genes encoding trithorax group protein, Appr1 processing enzyme family protein, and 30S ribosomal protein were decreased by PEG treatment but increased after recovery (Fig 8). In addition, genes encoding MYB and zinc-binding proteins were up-regulated by PEG treatment, whereas auxin-related and *AP2* genes were down-regulated by PEG treatment (Fig 8). These results verified the accuracy and reliability of the transcriptome sequencing data, suggesting that these genes may function in drought response and they may be explored for alfalfa breeding with drought-resistance.

## Discussion

In the present study, we have provided detailed information on the physiological and transcriptome data of *M*. *falcata* under drought stress. It can be inferred that *M*. *falcata* exhibit stress tolerance mechanism under simulated drought stress. The increase in SOD, POD, and CAT activities (Fig 1), and conductivity and soluble sugar content after stress treatment, indicated that *M*. *falcata* possessed a better ROS scavenging ability to resist drought. It was shown that the increase in activity of these enzymes could eliminate ROS and peroxides induced by stress and in turn inhibit the peroxidation of the plasma membrane, and protect cells from damage [38–40]. Our results suggest that *M*. *falcata* regulates the activity of defense enzymes to reduce the accumulation of harmful substances.

In this study, we also performed transcriptome sequencing and identified 255,014 unigenes in *M*. *falcata* (S2 Table). The N50 length of the unigenes was 1,026 bp, and the average length was 654 bp. These results were comparable to those obtained in recently published transcriptomic analyses of other plant species, such as *Reaumuria soongorica* (N50 = 1,109 bp, average length = 677 bp) [41] and Litchi (N50 = 811 bp, average length = 601 bp) [42]. In addition, more than 80% of the identified unigenes (220,137, 86.32% of the total) were successfully annotated using BLAST searches with the public Nr, Nt, Swiss-Prot, GO, COG, and KEGG databases. This is a considerable proportion, given the absence of genomic information on *M*. *falcata* (S7 Table). In the NR database, 75.2% of the gene function was annotated. The most common KOG annotation was “general function prediction only”, and this result was consistent with previous studies on alfalfa [43], indicating the great potential for gene function exploration in this plant species. Among these seven databases, 86.32% of the sequences were successfully annotated in at least one database. This means that 13.68% of the obtained gene sequences were not annotated, suggesting that these sequences may be associated with unique physiological processes or stress resistance mechanism in *M*. *falcata* (S7 Table). However, incomplete annotation may also result from technical limitations such as low sequencing depth or short read length as reported in other studies [44].

In this study, high-throughput sequencing data was used to characterize the transcriptome of *M*. *falcata*, the genomic data of which is currently unavailable. Our study provided the first fully characterized physiological, transcriptome and a comprehensive foundation for future molecular studies on *M*. *falcata*. In many studies, digital gene expression sequences could be mapped to the assembled transcriptome for further gene expression analysis. However, transcriptome sequencing results should be verified by qPCR, as was performed in a recent study for *Oxytropis ochrocephala Bunge* [45]. Many candidate genes involved in drought stress were identified from the RNA-seq data, and we also verified differential expression of 7 specific genes related to drought stress using qPCR. It has long been known that extensive changes in gene expression, including both up-regulated (456 genes) and down-regulated (250 genes) key genes (Fig 4B), occur when plants are exposed to drought stress as in other studies [46,47]. These results suggested that plants varied in their abilities to adapt to drought stress. In extreme cases, gene expression in desert plants may differ extensively from those in inland plants, and differences in gene expression patterns may confer enhanced ability for desert plants in response to drought stress.

In addition, drought-related genes are highly expressed, possibly through increased binding of specific transcription factors (ERF/AP2 and MYB) [48–51] (Fig 6) to improve drought tolerance, and this finding agrees with previously published works showing that the over-expression of AP2 transcription factor *NtERF172* conferred drought resistance [51]. Moreover, alcohol dehydrogenase involved in catalytic decomposition of ethanol, which is eventually oxidized into CO_2_ and H_2_O, providing an intermediate product for plant metabolism, eliminate the toxic effects of aldehydes [52], and reduced the toxicity of aldehydes under drought stress [53]. In our study, the gene encoding alcohol dehydrogenase was up-regulated, therefore, it could be the key gene in response to drought stress in *M*. *falcata*.

Moreover, GO and KEGG enrichment analyses on DEGs revealed that a number of differential expressed genes were most strongly enriched in biological processes, including citrate cycle (TCA cycle), fatty acid metabolism, carbon fixation in photo synthetic organisms and ribosome, enzymes distributed with a large proportion (S9 Table and Fig 5), indicating a variety of metabolites were synthesized *via* active metabolic processes in leaves of *M*. *falcata*. It has been demonstrated that powerful capability of RNA-Seq technique in identification of novel genes from non-model organisms, and these annotations also provided a valuable resource to study specific processes, and pathways involved in various plant growth and development as in other plant species [54].

Significantly enriched pathways identified by KEGG enrichment analysis include the phenylpropanoid biosynthesis, glycolysis/gluconeogenesis and flavonoid biosynthesis (S9 Table). These important pathways were also found to be significantly regulated under drought resistance in rice [55,56]. Our results also showed that drought stress affected plant respiration, enzyme activity, and metabolic-related processes. It was also revealed that phenylpropanoid biosynthesis was enriched during drought treatment in our study, which was consistent with another research [57,58]. Actually, phenylpropanoids with the greatest potential to reduce ROS were almost exclusively synthesized in response to a plethora of drought stresses [59]. In other studies, plants have resisted external pressures caused by drought stress by increasing antioxidant enzyme (SOD, POD, CAT) activity [60], POD is an essential antioxidant enzyme for plants and it acts synergistically with SOD, CAT, glutathione peroxidase, etc. in a variety of crops to remove harmful free radicals under adverse conditions. Interestingly, six enzymes annotated as peroxidase (POD) of the phenylpropanoid biosynthesis pathway (*P*<0.05) were up-regulated in treatment when compared to control (Fig 7B). In particular, the increased tolerance to drought stress is related to the enhanced phenylpropanoid pathway, which thereby could increase activity of antioxidant enzymes and reduced membrane damage [8]. Our results demonstrated that *M*. *falcata* also possesses a ROS scavenging system that responds to drought stress. The changes of POD reported here were consistent with an increased concentration of antioxidant enzymes, enabling plants to respond to stress and further to adapt to changes in external environment, and this is consistent with increased level of POD (Fig 1D).

The glycolysis/gluconeogenesis pathway mainly affected ATP supply to respond to abiotic stress [61]. In our study, pyruvate kinase was significantly up-regulated in the treatment and control groups, which is one of the main rate-limiting enzymes in glycolysis (Fig 7A). During glycolysis, pyruvate kinase can convert ADP into ATP, it is presumed that increase in expression level of *PK* gene may have produced more energy via the glycolysis pathway to tolerate drought [13]. It was reported that accumulation of pyruvate in glycolysis pathway has great impacts, and pyruvate decarboxylase (PDC) controls the anaerobic fermentation of pyruvate. In our study, expression level of *PDC* gene was significantly up-regulated under drought, therefore, increase of PDC in *M*. *falcata* under drought stress not only ensure continuity of the glycolysis process, but also consumes NADH produced during glycolysis process, to avoid cells be damaged by acidification. In summary, efficient up-regulation of genes of entire anaerobic metabolic machinery is essential to provide tolerance against drought [13].

In recent years, increasing research in molecular mechanism were focused on plant responses to drought stress, including studies on gene expression and signaling pathways. It becomes a critical breeding objective to search for drought resistance related genes from *M*. *falcata* and apply them in the improvement of alfalfa productivity in many drought-prone environment; it is of great theoretical and practical significance to dissect drought tolerance mechanism in *M*. *falcata*. In alfalfa, various regulatory mechanisms were developed to deal with drought, and to avoid or minimize cellular damage caused by drought stress, including higher proline, soluble sugar, malondialdehyde, SOD, POD, CAT content accumulation [38,62]. Furthermore, sequencing strategy was also used to identify drought-responsive microRNAs in alfalfa root and leaf tissue [63]. By analyzing the response of *M*. *falcata* to drought stress during different stress time, we have detected two significantly enriched pathways, together with 5 genes that are associated with drought resistance. It was evident that phenylpropanoids and glycolysis may play a role in countering drought-induced oxidative damage. This function may be of great value when key components of the highly coordinated network of antioxidant defense are impaired. In addition, the candidate genes can be used as a basis in molecular breeding to improve drought resistance and yields of alfalfa.

## Conclusion

In conclusion, we reported the physiological and transcriptome changes in *M*. *falcata* under drought treatment and recovery. First of all, our results revealed the relationship between physiological and transcriptome changes under drought stress in *M*. *falcata*. Besides, gene enrichment analyses on differentially expressed genes revealed that phenylpropanoid biosynthesis (ko00940) and glycolysis/gluconeogenesis (ko00010) were the two key pathways involved in drought response in *M*. *falcata*. Finally, the expression levels of seven DEGs were verified to be involved in drought response in *M*. *falcata* by qPCR analysis. Taken together, these results will provide valuable information related to drought stress response in *M*. *falcata* and lay a foundation for molecular studies and genetic breeding of legume crops in future research.

## Supporting information

S1 Fig Statistics analysis of nonredundant (nr) peptide database. A BLASTx comparison of the *M*. *falcata* transcriptome to NCBI nonredundant (nr) peptide database showing percentage of hits with other plant species.
(TIF)

S2 Fig Twenty most highly expressed DEGs in each combination aligned with FPKM value. (A) treatment vs control. (B) recovery vs treatment. (C) recovery vs control. Red indicates low levels of expression and green indicates high levels of expression.
(TIF)

S3 Fig Relative expression level of 10 *POD* genes and one *SOD* genes. *POD* and *SOD* genes that were differentially expressed in the control, PEG-treatment and recovery groups as shown in heatmap. Color from blue to red indicate the expression level from low to high.
(JPG)

S1 Table Sequences of primers used for qPCR. (XLSX)

S2 Table Data output quality. (XLSX)

S3 Table Number of transcripts/unigenes of different length. (XLSX)

S4 Table Analysis of transcripts/unigenes length. (XLSX)

S5 Table Analysis of FPKM. (XLSX)

S6 Table Analysis of percentage of gene annotation. (XLSX)

S7 Table Number and proportion of genes in *M*. *falcata* for NR comparison with other plant species. (XLSX)

S8 Table GO classification of unigenes from *M*. *falcata*. (XLSX)

S9 Table KOG classification of unigenes from *M*. *falcata*. (XLSX)

S10 Table KEGG classification of unigenes from *M*. *falcata*. (XLSX)

S11 Table Significant GO enrichment terms for identified DEGs. (XLSX)

S12 Table Significant KEGG enrichment analysis of DEGs. (XLSX)

S13 Table Number of transcription factor genes in *M*. *falcata*. (XLSX)

S14 Table Details information of transcription factor genes that were differentially expressed. (XLSX)

S15 Table The DEGs related to phenylpropanoid and glycolysis/gluconeogenesis pathway. (XLSX)

S16 Table Information of DEGs verified by qPCR. (XLSX)
