# Supplementary figures and images for: Physiological and transcriptome analyses highlight multiple pathways involved in drought stress in Medicago falcata
Source: PLoS One. 2022 Apr 7;17(4):e0266542. doi: 10.1371/journal.pone.0266542 (PMC8989214; doi:10.1371/journal.pone.0266542)

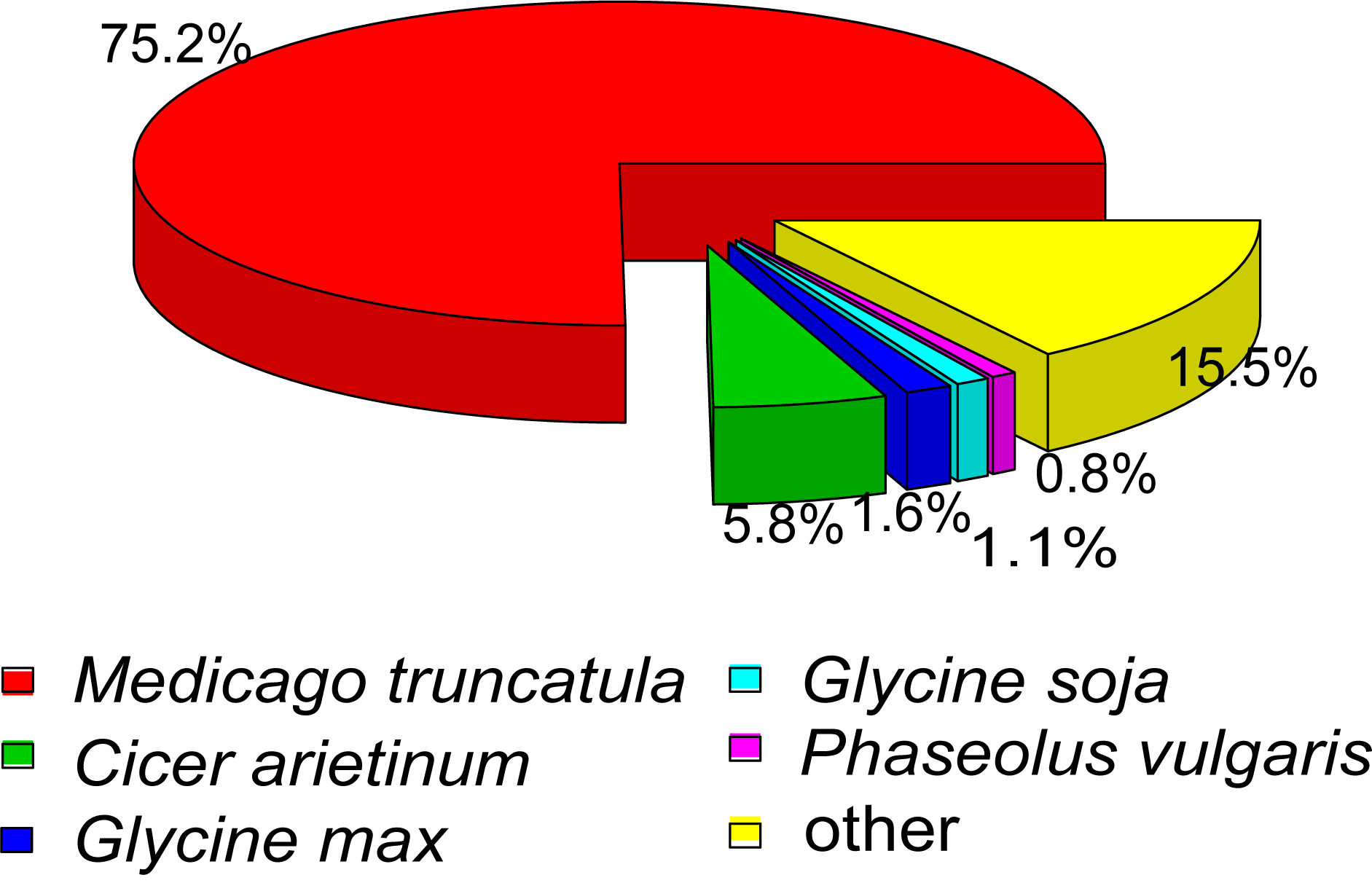

Supplement: S1 Fig — A BLASTx comparison of the M. falcata transcriptome to NCBI nonredundant (nr) peptide database showing percentage of hits with other plant species. (TIF) [file pone.0266542.s001.tif]

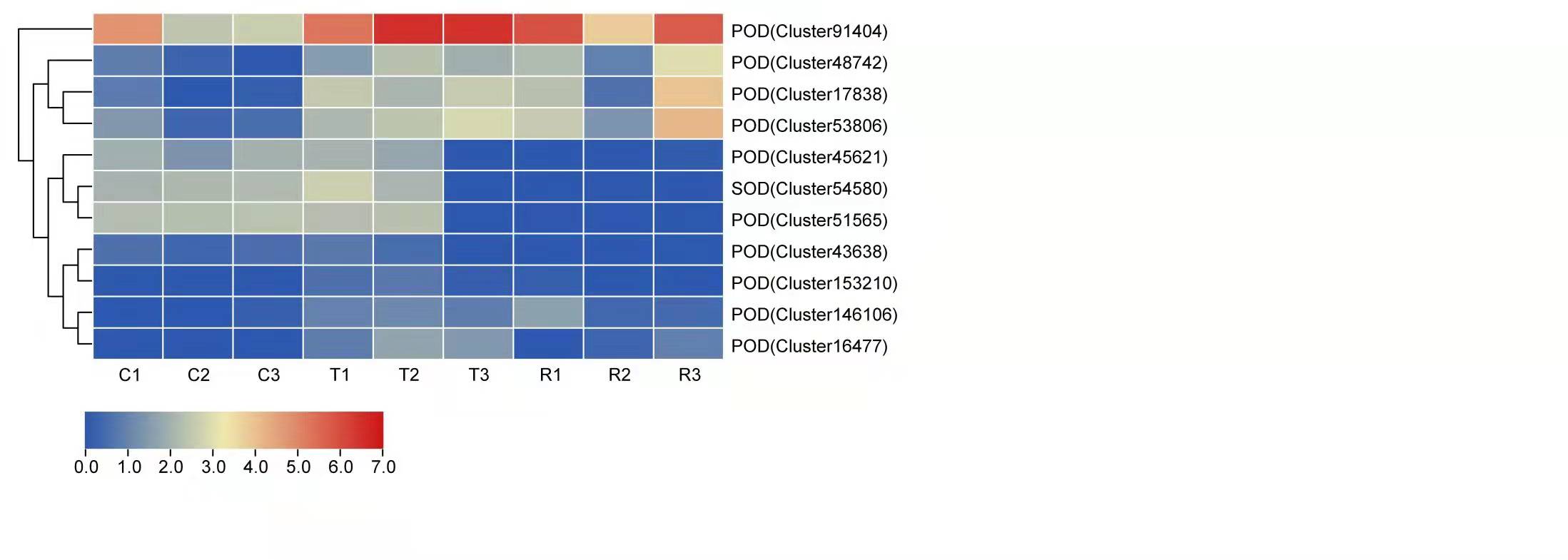

Supplement: S3 Fig — POD and SOD genes that were differentially expressed in the control, PEG-treatment and recovery groups as shown in heatmap. Color from blue to red indicate the expression level from low to high. (JPG) [file pone.0266542.s003.jpg]
